# Communicating Risk for Obesity in Early Life: Engaging Parents Using Human-Centered Design Methodologies

**DOI:** 10.3389/fped.2022.915231

**Published:** 2022-06-28

**Authors:** Erika R. Cheng, Courtney Moore, Lisa Parks, Elsie M. Taveras, Sarah E. Wiehe, Aaron E. Carroll

**Affiliations:** ^1^Department of Pediatrics, Division of Children's Health Services Research, Indiana University School of Medicine, Indianapolis, IN, United States; ^2^Indiana Clinical and Translational Sciences Institute (CTSI), Research Jam, Indianapolis, IN, United States; ^3^Kraft Center for Community Health, Massachusetts General Hospital, Boston, MA, United States; ^4^Department of Nutrition, Harvard T.H. Chan School of Public Health, Boston, MA, United States; ^5^Department of Pediatrics, Center for Pediatric and Adolescent Comparative and Effectiveness Research, Indiana University School of Medicine, Indianapolis, IN, United States

**Keywords:** obesity prevention, parents, human-centered design (HCD), communication, early life

## Abstract

**Objective:**

Pediatricians are well positioned to discuss early life obesity risk, but optimal methods of communication should account for parent preferences. To help inform communication strategies focused on early life obesity prevention, we employed human-centered design methodologies to identify parental perceptions, concerns, beliefs, and communication preferences about early life obesity risk.

**Methods:**

We conducted a series of virtual human-centered design research sessions with 31 parents of infants <24 months old. Parents were recruited with a human intelligence task posted on Amazon's Mechanical Turk, via social media postings on Facebook and Reddit, and from local community organizations. Human-centered design techniques included individual short-answer activities derived from personas and empathy maps as well as group discussion.

**Results:**

Parents welcomed a conversation about infant weight and obesity risk, but concerns about health were expressed in relation to the future. Tone, context, and collaboration emerged as important for obesity prevention discussions. Framing the conversation around healthy changes for the entire family to prevent adverse impacts of excess weight may be more effective than focusing on weight loss.

**Conclusions:**

Our human-centered design approach provides a model for developing and refining messages and materials aimed at increasing parent/provider communication about early life obesity prevention. Motivating families to engage in obesity prevention may require pediatricians and other health professionals to frame the conversation within the context of other developmental milestones, involve the entire family, and provide practical strategies for behavioral change.

## Introduction

Overweight and obesity now affect over 41 million children under the age of 5 (1), an epidemic that is apparent worldwide (2, 3). Childhood obesity negatively impacts health (4–11) and is known to track along the life course (12, 13), increasing the risk for many related adult diseases (14). As such, prevention is key, with early life holding the most promise for addressing the obesity epidemic (15).

Pediatricians can help curb the obesity epidemic through screening, communication, and anticipatory guidance. Beginning at age two, the American Academy of Pediatrics (AAP) recommends measuring BMI and screening for obesity-related comorbidities as part of routine primary care, but research indicates that parents (16) and providers (17, 18) often avoid the subject. In addition to time and resource constraints, providers report feeling uncomfortable about raising the issue, uncertainty about how best to communicate with families about weight, and concern about how families will respond (19). Obesity counseling in infancy may be even more challenging – despite evidence of modifiable risk factors (14) – because parents may be unaware of the importance of prevention and because there is no standard recommendation for treatment of overweight infants.

To inform the development of a communication strategy focused on helping pediatricians and parents of young children engage in conversations about obesity prevention, we employed a human-centered design (HCD) approach to gain a richer qualitative understanding of parents' perceptions, concerns, beliefs and communication preferences about early life obesity risk. HCD, which is increasingly being used within healthcare, is a generative, iterative process that includes: (1) insight, or developing an understanding of stakeholders and their needs; (2) ideation, or exploring options for “what could be”; and (3) implementation, or executing the intervention and testing prototypes (20). Additional core principals of HCD include developing deep empathy with end users and acknowledging lived experience as a credible form of expertise (21). As such, HCD is particularly effective in eliciting deep insights and creating solutions compatible with end stakeholders' needs (22) and may help broaden our understanding of factors not addressed by traditional qualitative approaches that tend to engage with participants in a consultative, rather than a collaborative manner (23). HCD shares many theoretical underpinnings of social behavioral research and implementation science; however, while these traditional approaches are useful to identify determinants of behavior change and evaluate how well a design solution works, HCD focuses on creating approaches and delivering solutions to problems based on a concerted effort to understand the specific needs and perspectives of users (24). HCD allows for iterative development and refinement of a customized and tailored solution, thereby increase the likelihood of user engagement. As such, HCD can help “move the needle” and galvanize new thinking around potential solutions intransigent problems (25). Previous work has shown that applying user-centered design can drive progress for health-related behavioral interventions (26).

HCD has been successfully used to create tools for enhancing communication interventions (27) but to our knowledge, has not been applied to elicit parent perspectives of early life obesity risk. We hypothesized that a HCD approach could identify key views of obesity risk and parental preferences regarding risk communication for young children.

## Methods

### Study Design

We collaborated with the Indiana Clinical and Translational Sciences Institute's Patient Engagement Core (28), Research Jam (RJ), a multi-disciplinary team of trained HCD experts and health services researchers with experience applying HCD techniques to health services research through a combination of qualitative research and design research methodologies (29–31). Study protocols were approved by the Indiana University Institutional Review Board.

We (CM, LP, and ERC) conducted a series of online HCD activities with parents of children <24 months of age that focused on exploring underlying attitudes, beliefs, and communication preferences related to early life obesity. All activities took place virtually on FocusVision's Revelation (32) a user-friendly, online qualitative research platform that allows researchers to craft activities for participants to complete using their personal computer or smart phone. Using social media-style interactions, Revelation strives to engage participants in activity-based research, enabling them to share their thoughts, feelings, photographs and video, all of which deliver rich, deep insights into their lives.

### Parent Recruitment

RJ recruited parents between July and December 2020 via: (1) a Human Intelligence Task (HIT) posted on MTurk, a large, online crowdsourcing marketplace frequently used for survey studies (33); (2) social media; and (3) local professional organizations our University has partnered with in previous work.

***Amazon's Mechanical Turk (MTurk)*** identifies online “workers” who meet specific, predefined criteria and alerts them of HITs to complete for compensation. Workers read brief descriptions and preview the tasks before working on them. MTurk allows researchers to imbed prescreening questions that constrain who can see and complete particular HITs. We programed our HIT to reach workers who: (1) were at least 18 years old; (2) lived in the US; (3) had least 1 child <24 months of age; and (4) were able to read and write in English. Parents who completed the survey were reimbursed through Amazon's pre-determined fee schedule.

We also posted study information on various ***social media*** platforms, including Facebook (parenting groups, RJ and Indiana CTSI pages), and on the sub-Reddit r/parents.

Finally, we shared a study flier with ***local community organizations*** who serve parents, including the IUPUI Center for Young Children, Tabernacle Presbyterian Church in Indianapolis, the local office of the Women, Infants, and Children (WIC) Program, the Indiana State Department of Health, and Indiana CTSI coalitions.

Through an electronic link or QR code, we directed parents to a RedCap page containing an overview of study activities. Interested parents were screened for eligibility, provided an electronic consent, and completed a brief demographic survey. We selected a subset of these parents to ensure diversity of child age, income, and mother/father role on a first come, first serve basis. We then emailed these parents full study details, instructions, and a link to FocusVision's Revelation webpage.

### Human-Centered Design Activities

Parents then engaged in a series of online activities designed by RJ focused on perceptions of parenting advice, health in infancy, obesity prevention, and communication preferences. To facilitate discussion, we grouped parents based on child age (<12 months or 13–24 months old) and household income (below or above $50,000). Activities consisted of three open-ended questions focused on parenting and obesity, and three personas/empathy exercises followed by discussion. These activities were designed based loosely on a health-belief model (34) which assumes that parental involvement in childhood obesity prevention is partly determined by their perception of the likelihood, seriousness, and potential consequences of their child's obesity risk. Parents performed activities asynchronously throughout a week-long engagement period. We monitored their progress, asked follow-up questions, and sent reminders to complete the activities when needed. Parents spent approximately 1.5 h to complete the activities and were compensated $50. Revelation activities took place during the spring of 2021.

#### Open-Ended Questions

The open-ended questions were completed individually; the first two questions were shared so others in the group could respond and discuss. The questions were developed to gain an understanding of perceptions around health and healthy behaviors for infants and toddlers (“What do you think is most important for making sure your baby is healthy?”); to understand what kinds of unsolicited advice parents get in their daily lives, how the advice is received, and factors impacting how advice was received (“Tell a story about a time you were given parenting advice that you didn't ask for. What was the advice and what was your reaction?”); and to elicit underlying attitudes around obesity (“Define obesity in your own words. What other words do you associate with obesity?”).

#### Personas/Empathy Exercises

Personas (35) (e.g., fictitious representations of a person used to capture important characteristics and build empathy) and empathy maps (36) (e.g., a structured tool used to build empathy for an individual by thinking about what they say, think, feel, and do in a particular situation) are techniques used in HCD to help designers build empathy with the “customer” for whom they are designing. We developed three individual persona and empathy map exercises within Revelation that explored parents' beliefs and communication preferences about obesity and overweight at different periods in the life course. Each persona displayed a stock image of someone with unhealthy weight, an exercise prompt, and a series of open-ended questions for parents to complete on their own time. The subject of the persona became increasingly younger in each series, such that the final persona was focused on a baby with an unhealthy weight. Specific components of the activities are shown in Table 1. Parents then convened as a group to reflect on the activity.

**Table 1:** Human-centered design activities completed in revelation: persona and empathy map activities, discussion prompts, and group discussion questions.

**Persona**	**Bob**	**Alexis**	**Friend's Baby**
Image	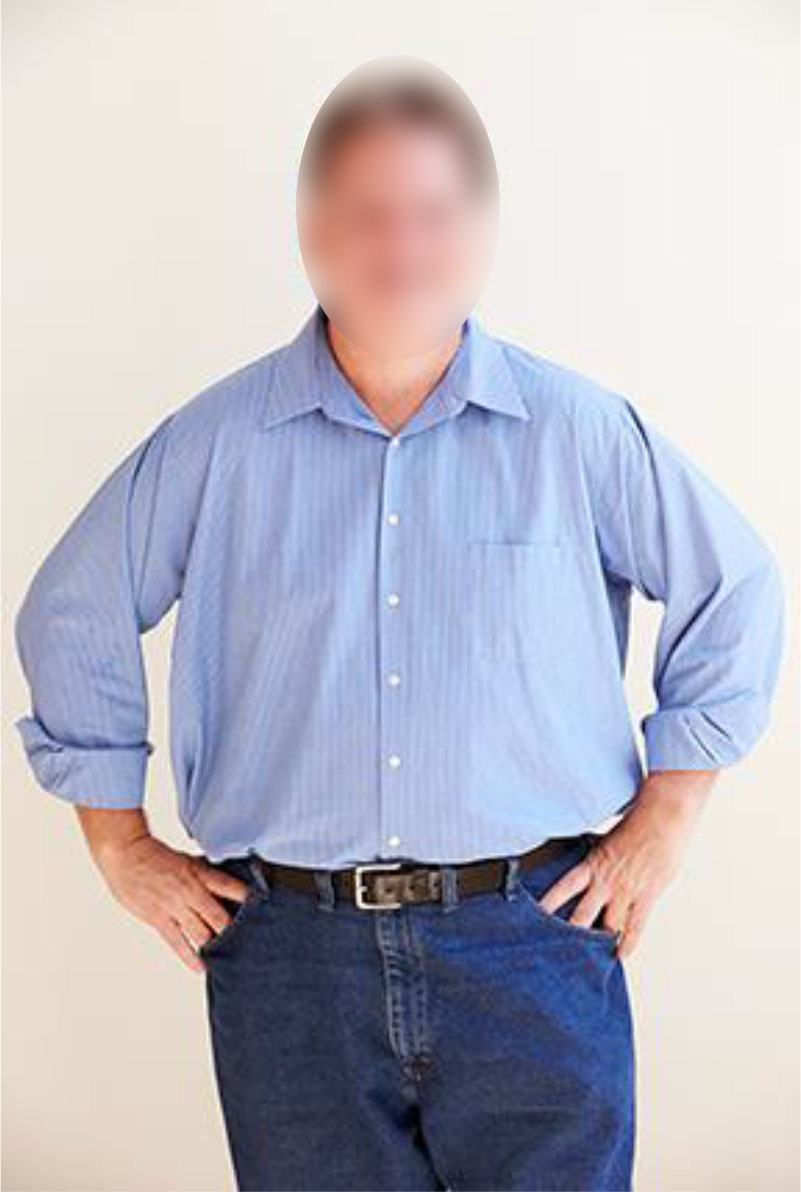	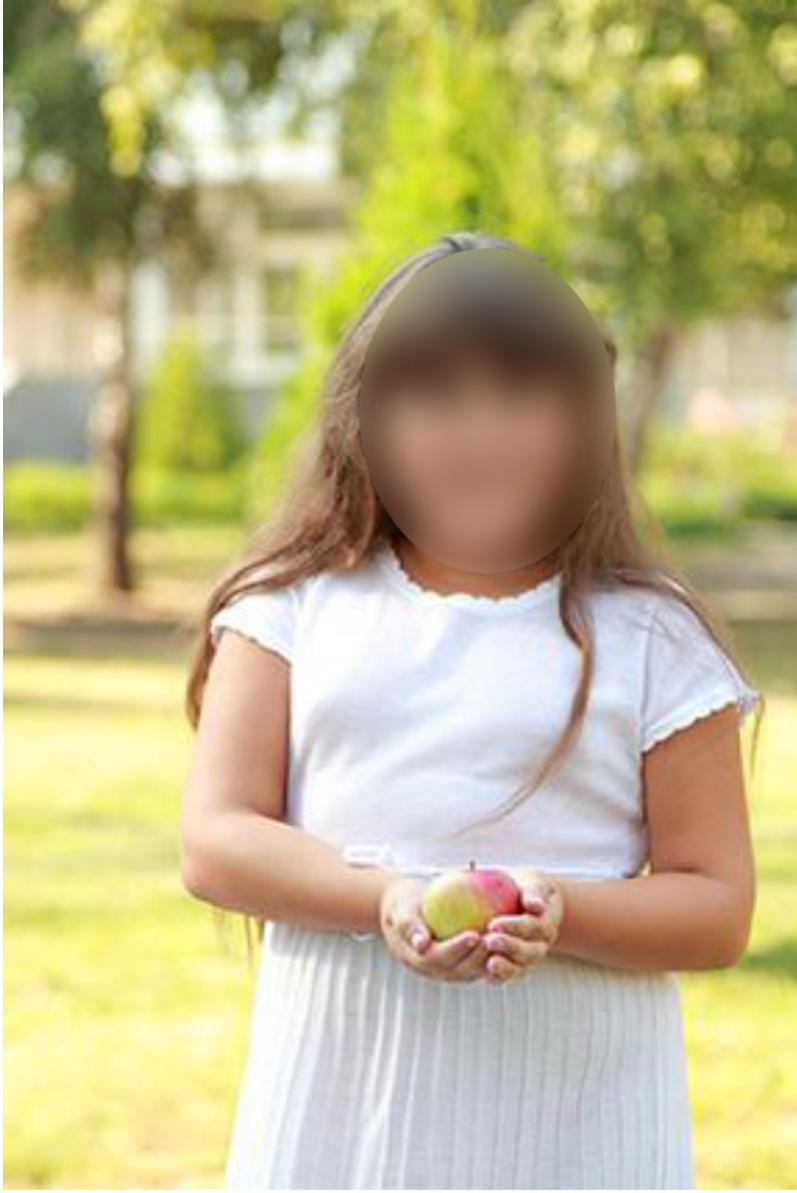	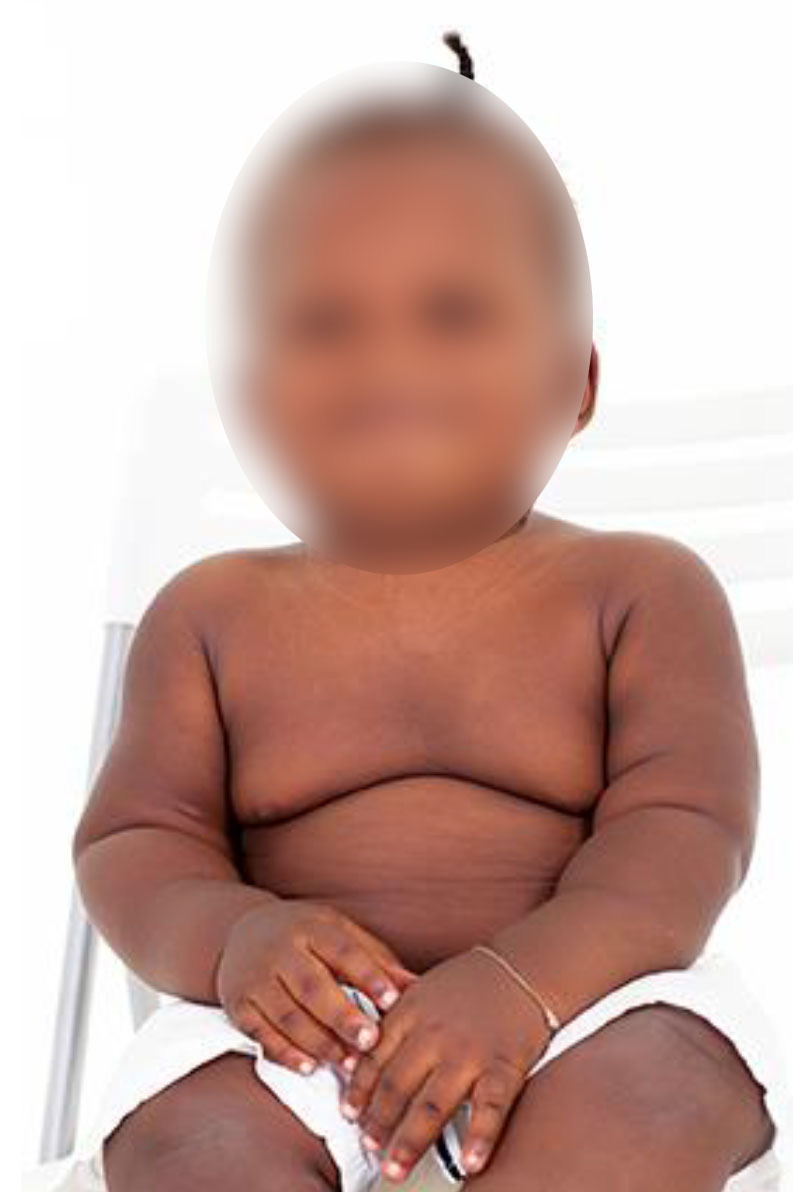
Prompt	This is Bob. His weight is unhealthy. You care about Bob and want him to be healthy. You wish someone would talk to him about his health.	This is your niece, Alexis. Her weight is unhealthy. You care about her and want her to grow into a healthy adult. You wish someone would talk to Alexis and her parents about her health.	This is your friend's baby. The baby's pediatrician has said his weight is unhealthy. Your friend isn't concerned about the baby's weight.
Open-ended questions	1. Would you be worried to talk to him yourself? Why or why not? 2. Who do you think is the best person to talk to Bob? What do you think this person should say or do to encourage him to be healthier? 3. How do you think Bob might react? 4. What changes should Bob make in his life to get to a healthier weight?	1. Would you be worried about talking to Alexis yourself about her weight? Why or why not? 2. Who do you think is the best person to talk to Alexis about her weight? What do you think this person should say or do to encourage her to be healthier? 3. How do you think Alexis might react? 4. Who do you think would be the best person to talk to Alexis's parents about her weight? What do you think this person should say or do to encourage Alexis's parents to think about her weight? 5. How do you think her parents might react? 6. What changes could Alexis make to get to a healthier weight?	1. Do you think your friend should be concerned about the baby's weight? Why or why not? 2. What changes could your friend make to get the baby to a healthier weight? 3. Would you be worried about talking to your friend about the baby's weight. Why or why not? 4. Who do you think is the best person to talk to your friend about this? What should this person say or do to encourage your friend to think about the baby's weight? 5. How do you think your friend might react?
Group discussion questions	1. What was it like to complete the Bob activity? 2. What challenges did you have?	1. What was it like to complete the Alexis activity? 2. What was challenging about it? 3. How did it compare to completing the Bob activity?	1. What was it like to complete the Friend's Baby activity? 2. What was challenging about it? 3. How did it compare to completing the Bob and Alexis activities?

### Data Analysis

Two members of RJ independently coded responses from four parents (one per group) as well as all four Friend's Baby discussions. The four parents were chosen because they had a high completion rate and thorough answers. The coders allowed codes to emerge from the data and then met to refine their coding structures. The coders then divided the remaining discussions and responses and coded them using the final coding structure.

## Results

### Session Participation

From a pool of 163 parents who consented to the study, we invited 63 to participate in Revelation after excluding invalid and incomplete responses; 31 participated and 27 (87.1%) completed all of the activities (Table 2).

**Table 2:** Sample characteristics.

**Sample characteristics**	***N*** **(%)**
**Relationship to child**	
Mother	26 (83.9)
Father	5 (16.1)
**Marital status**	
Married	22 (71.0)
Never married/divorced	9 (29.0)
**Child age**	
0-12 months	16 (51.6)
13-24 months	15 (48.4)
**Annual household income**	
< $50,000	9 (29.0)
≧$50,000	22 (71.0)
**Race/ethnicity**	
White	21 (67.7)
Black	5 (16.1)
Asian	2 (6.5)
Hispanic/latnix	3 (9.7)
**Highest education**	
Highschool or less	3 (9.7)
Some college	7 (22.6)
Bachelor's degree	13 (41.9)
Graduate/professional degree	8 (25.8)

### Session Findings

#### Parental Views About Obesity and Obesity Risk

Seven overarching themes emerged from the persona/empathy map exercises. Table 3 presents additional statements from parents.

**Table 3:** Parental views about obesity and obesity risk in the first 1,000 days: additional statements from the persona/empathy map activities.

**Obesity is more than just weight**	*Obesity is when someone is so overweight it starts to noticeably affect their health and quality of life*.
	*I do think that for most people, obesity (and even those overweight who are not technically obese) causes a number of additional problems, whether seen or unseen, some of which won't manifest in obvious ways or until later*.
**Anxiety and Stigma**	*I think it's important not to make assumptions about someone and to remember [that] everyone has different body shapes*.
	*Not everyone is supposed to be “thin” or “skinny” – we all have different shapes*.
	*I think weight is so entangled in harmful social messages (e.g., if you are overweight, you are lazy, gross, bad, etc.) that it can be hard to talk about weight-loss productively at all – except in very safe spaces where the person will feel vulnerable but not attacked or judged*.
	*Young girls are so impressionable, I feel as though discussions regarding their weight should be handled delicately. One false move and you might traumatize a child*.
	*Male-defined ideals of feminine beauty/body type are already ingrained in our society and negatively impact girls' health, so I would not want to inadvertently exacerbate these toxic messages by having a conversation about [Alexis'] weight/body*.
**Obesity is more impactful at older ages**	*No, I don't think a baby being at a higher weight is a big deal. I feel that…it's of little consequence later in life*.
	*I don't think I would be overly concerned about the baby having a bigger weight in the beginning*.
**Pediatricians are important sources of health information**	*I would likely listen to the pediatrician's recommendation simply because they have dealt with the baby weight issues more than I have and if a doctor is concerned, I tend to be concerned too and I think my friend should feel the same*.
**Uncertainty about risk and management of obesity in infancy compared to older ages**	*My first was all over the chart in his first year starting in the 90th percentile at birth down to the 1st percentile at 4 months. He was growing, and his doctor wasn't con-cerned at any point. By a year he evened out in the 60th-70th percentile. There is such a wide range of normal weights in the first year, I wouldn't be concerned unless the pediatrician was concerned*.
	*I can relate to this now that I'm reading this perspective. This baby may be overweight, but as long as it fluctuates and he is developing on track, that seems like the MOST important part. Maybe down the line if the baby seems to be going up and up on the weight charts it could be looked at as a more significant trend and not just a spurt or insignificant weight increase*.
	*Honestly, when it comes to baby's [sic] and “losing weight” that does not seem right to me*.
	*Most babies carry weight. A lot of time it's a good thing. It shows the baby is eating well and getting their nutrients. There are not able to move around as much as toddlers or adults*.
	*This [Friend's Baby] activity was more challenging for me because I feel more knowledgeable about the weight of adults or older children than I do of babies*.
**More concern about developmental delays and underweight**	*If obesity makes baby barely willing to crawl or stand, or sit, and makes it harder for the baby to crawl or sit then it is definitely a big deal*.
	*I think if caregivers are educated on healthy diet …then higher weight is not a big deal. I would be more concerned about a baby being underweight or not gaining weight because that can play a role on development*.
**Parents know best**	*My child turned a year old recently and I am still breastfeeding on demand. Several individuals have [told me] that I should wear her immediately to prevent stunts in her development and break her attachment to me because she prefers mommy to everyone else*.
	*My in-laws have tried to give us plenty of advice. For example when my baby was running a fever they tried to tell me to put Gatorade in his bottle. This is something I would never do*.
	*I don't let my kids just go into my kitchen and grab whatever they want. They have to ask me first. I was told to not treat my kids like “they were in jail”. The person that said that only had one child which its totally different with many kids. At first I was highly offended because I would never mistreat my kids. I simply explained that I am on a tight budget and my kids like to eat up everything just cause it's there not cause they are hungry. In order to make sure my kids don't eat up everything and upset their stomach in the process I have them ask me first and most the time it's a yes. After explaining that the person didn't realize how it was having many kids and apologized for their comment*.
	*One piece of advice I seem to get a lot of is about how to help my daughter “eat more”. She is a very picky eater and my husband and I make sure she gets what she needs every day and have plan we work on. But, I think people on the outside don't always understand that and always offer tips or trades or comment on how little she eats and how small she is. I try not to take offense because for the most part I do think they are trying to help. I do get more offended when they comment that she is so tiny, but I think that is my insecurity more than anything*.

##### Perception That Obesity Is More Than Just About Weight

A majority of parents defined obesity as having a high weight for length in addition to associated health problems or negative impacts to quality of life. They were less concerned about body mass index alone.

- *[Obesity is] the point when it's no longer just carrying an extra 30-50 pounds [but] when it starts to seriously hinder a person's quality of life and health*.
- *I think it's possible to be technically obese by the numbers (though I believe that BMI is outdated) and still be as healthy (or healthier) than someone of average weight*.

##### Anxiety and Stigma

A common sentiment expressed by parents during the Bob Activity was anxiety about labeling:

- *The questions made me have to assume things about Bob based on the way he looks, so I didn't want to assume*.

Societal stigma was apparent in the discussions. Parents were concerned that conversations about weight would harm Bob and Alexis and were especially hesitant to discuss Alexis's weight for fear of causing trauma.

- *Even though I am worried about [Bob's] health, he might feel like I'm attacking his appearance or even his character because being obese is viewed by society as an indicator of ugliness and laziness*.
- *No one (even a family member) should be sitting down with Alexis and talking about her weight. There are just already too many toxic messages about young girls and their bodies/weight. – I would not want to set her up for future eating disorders or something else*.

##### The Belief That Obesity Is More Impactful at Older Ages

Parents did not challenge the idea that a conversation around infant weight was appropriate, but were less concerned about obesity risk in early life than during the Bob and Alexis activities. Parents viewed early habits as easier to change.

- *If there is a noticeable problem by age 3 or 4 then an intervention might be necessary*.
- *I did find myself not concerned whatsoever with the weight of the baby, slightly concerned with the weight of Alexis, and most concerned with the weight of Bob*.

##### Pediatricians Are Important Sources of Health Information

Parents expressed during the Friend's Baby Activity that they would take weight-related concerns by their child's pediatrician seriously.

- *If the baby's pediatrician has expressed concern about the baby's weight, then I would definitely be worried. A medical professional's opinion on weight is important to consider – they have the baby's health as their primary interest – so I trust their perspective*.

##### Uncertainty About Risk and Management of Obesity in Infancy Compared to Older Ages

When discussing whether infants could have overweight, parents believed that infant weight fluctuations are normal and that most infants grow out of weight issues.

- *This baby may be overweight, but as long as it fluctuates and he is developing on track, that seems like the MOST important part. Maybe down the line if the baby seems to be going up and up on the weight charts it could be looked at as a more significant trend and not just a spurt of insignificant weight increase*.

Some parents expressed that “baby fat” is not concerning, or was a sign of a healthy baby. They expressed that once a baby begins moving more, the weight would naturally drop off.

- *I don't think I would be overly concerned about the baby having a bigger weight in the beginning. So many babies lose their “baby fat” once they become mobile and really get into walking, climbing, jumping, and dancing*.

Parents were confident of the causes and long-term impacts of obesity during the Bob and Alexis activities, and had opinions and recommendations for lifestyle changes that could improve their health, they were less knowledgeable during the Friend's Baby activity.

- *I am less clear on what the healthy standards are for decreasing weight in babies, so I don't feel qualified to give advice in this area*.

##### More Concern About Developmental Delays and Underweight Than Overweight and Obesity

Similar to findings from the Bob Activity, parents expressed that infant weight itself is not concerning unless there are associated health problems, and placed a higher value on attributes such as childhood happiness: *most importantly, we want him to be healthy and feel good* and meeting developmental milestones. They expressed more concern about having a baby with underweight than with overweight.

- *If it were my child, I would start to be concerned if my baby was healthy and also seemed to be abnormally fixated on food or if they were missing gross motor milestones because it was hard to move themselves around*.
- *When I think about babies and weight, my mind initially goes to a baby not gaining enough weight; I do not ever [think about] a baby being overweight*.

##### Parents Know Best

Parents shared many examples of unsolicited advice they had received about their child's diet or eating habits, but emphasized that they knew best when it comes to their own children.

- *Every child is different and you just have to find out what works for yours. I don't take offense when people try to give me advice because maybe it's what worked for them but I also take into account that I know my children better than anyone*.

#### Parental Preferences Regarding Early Life Obesity Risk Communication

Parents were also asked about their preferences in a health professional approach to obesity risk communication. Eight key themes emerged from their answers (Table 4).

**Table 4:** Parental preferences regarding early life obesity risk communication.

**Build rapport**	***Pediatricians should have established a good rapport with a family before discussing diet – definitely not a first-visit conversation***.
	*I think pediatricians really have to come from a place of advice and education early and often during well baby visits so that parents expect that sort of support and coaching/counseling*.
**Listen first**	*Before the discussion takes place, the pediatrician should ask the parents to describe the food/feeding/activity level of the child. It's important to know what's really going on at home in order to know how to frame the conversation going forward*.
	*I feel it's most important for the physician or practitioner to fully understand the child and their diet before drawing conclusions or making specific recommendations because every family and child is so different. Pediatricians can usually offer parents great advice if they ask the right questions and receive honest answers from patients and caregivers. This requires strength in communications and diagnosis before even approaching a conversation about obesity*.
	*I also think pediatricians should really focus on asking about, supporting and helping parents troubleshoot around the habits that lead to a healthy weight over time (food choices offered and when/how many meals, beverage choices, active play vs screen time, etc.)*
**Make it a conversation, not a lecture**	*We, as parents, want the best for our children and if what is meant to be helpful advice comes across as accusatory, it can feel like we're not doing a good job. A discussion rather than a lecture from a pediatrician would be much more effective*.
	*I definitely think a good bed side manner is imperative. The pediatrician should not come across negative or condescending in any way*.
	*I think what I as a parent would like to hear is not that I am doing something wrong right now in this moment, but that I can take steps to positively influence my child's future and provide them a better life*.
	*Ultimately, making sure the parent feels confident and not belittled by the doctor will have the best effect*.
**Speak up**	*If the baby's pediatrician has expressed concern about the baby's weight, then I would definitely be worried. A medical professional's opinion on weight is important to consider – they have the baby's health as their primary interest – so I trust their perspective*.
	*For pediatricians, first of all, the key point is to make sure to parents that the baby's obesity is worth heeding. It is a big deal*.
**Make it a team effort**	*Also, if a pediatrician has a concern like the baby is overweight they should take in a positive way and bring up what to do to solve this problem as a team and not like it's all on the parents*.
	*If the parent is open to suggestions and change I would help them develop a plan of action in supporting change to lead to a healthier lifestyle*.
	*The pediatrician could frame it so that they (the Dr. and the parents) are on the same team, and they will work together give the child the best possible chance at health now and in the future*.
**Involve the whole family**	*I think that it is on the parents to set up a new environment to create a “healthy family” instead of just “work on Alexis's weight issues.” This re-framing ensures that Alexis herself is not deemed to be a problem that needs fixing. However, as I said at first, it is difficult for this change to occur if the parents refuse to change their own behaviors, especially those that could be negatively impacting Alexis (like stocking unhealthy snacks, leading very sedentary lifestyles, etc.)*.
	*[The Friend's Baby] would be an especially difficult conversation because it could not so easily be framed as a “this is an issue for the whole family.” However, I still think that this framing could be tried*.
**Discuss the long-term implications**	*I think the best approach is to talk about the other health issues that obesity can cause: heart conditions, diabetes, asthma. Helping the friend to see the long terms effects instead of the here and now might help the friend be more serious*.
	*I think it's also important to stress the health issues and complications that can arise from childhood obesity*.
	*Their pediatrician should inform them of the possible health risk if they do not improve the weight of their baby*
**Start with small, practical changes**	*I think for all of us guidance on how to tune into baby's cues and help figure out hunger from distress or boredom can be helpful especially in the toddler years*.
	*I also think meal suggestions would help. Most parents understand their child may be overweight but ideas on how to prepare kid friendly healthy meals might help*.
	*I also think pediatricians should really focus on asking about, supporting and helping par-ents troubleshoot around the habits that lead to a healthy weight over time (food choices offered and when/how many meals, beverage choices, active play vs screen time, etc.)*.
	*Doctors can help by providing helpful and easy to digest information for their patients, even if it is redundant. I may be in the minority here, but I would have loved our pediatrician to have provided a list of good snacks at certain ages ¬ in reality, we just googled “healthy snacks” and bought stuff that seemed healthy*.
	*Give the parents information on how to cook healthier meals and safe substitutions for sugars and carbs. People don't understand that eating rice is worse for your body than a baked potato*.

Parents repeatedly expressed that if their child's pediatrician was concerned about their child's weight, they would also be concerned (***Speak up***), but they preferred to have pediatricians build a strong relationship with them prior to discussing overweight in their infants (***Build rapport***). They also suggested that health professionals ask about the family's current habits before giving advice (***Listen first***). During the Bob and Alexis activities, the parents suggested that this approach could help identify barriers to healthy lifestyle changes (e.g., food insecurity or underlying medical issues), enabling targeted advice. To avoid defensive responses, parents recommended approaching the issue as a conversation rather than a lecture, and using language to help them feel encouraged and empowered, not judged (***Make it a conversation, not a*** ***lecture***).

Parents also suggested pediatricians frame conversations as a team effort between themselves and the parents, in part by offering direct support (***Make it a team effort***). Along those lines, parents suggested having family members join in the healthy lifestyle changes that Bob and Alexis were attempting (***Involve the whole family***). In this way, they could be supportive of the person needing to lose weight and also create a healthy family context within which to make these changes. Parents thought this would also be a better approach because it focuses on what “we” could do to be healthy instead of what “you” needed to do.

In terms of specific content, parents recommended framing the conversation around the long-term health implications of obesity to help them understand the seriousness of the issue (***Discuss the long-term implications of unhealthy weight***). Parents also agreed that habits were easier to change at younger ages and believed that small changes could make a big difference (***Start with small, practical changes so parents do not feel overwhelmed***). They identified specific areas of need, including resources to recognize fullness cues, ideas for active play, and healthy diet recommendations.

## Discussion

Using an online research tool called Revelation (32) and a HCD approach, we engaged parents of children <2 years of age in discussions around parenting, obesity, and communication about obesity and health. Our final sample included a diverse sample of 31 parents recruited based on household income and caregiving role. Results highlight the translational potential of using HCD to inform the design of a communication strategy that helps motivate families to engage in early life obesity prevention.

Our findings reinforce prior studies suggesting that parents are often not concerned about weight in infancy and tend to believe that children “grow out” of weight problems (37). Although parents welcomed a conversation about infant weight and obesity risk, there was a strong belief that BMI is not a useful measure for overweight and obesity in the absence of related health problems. Few parents perceived excess weight in infancy as an issue causing immediate health risks; rather, they were most concerned about infant weight when they perceived it to affect other aspects of development. Other research has similarly found that parents' immediate concerns relating to overweight were in relation to initiation of walking and other developmental milestones (38). Therefore, framing weight-related conversations in this context, as opposed to focusing on obesity or measures of weight-for-length, seems most likely to influence parental receptiveness to engage in preventative interventions.

At the same time, parents in our study overwhelmingly agreed that healthy habits are easiest to achieve earlier in life. While they were somewhat knowledgeable about obesity prevention and risk in older children and adults, our study also revealed gaps in knowledge regarding which habits in infancy lead to unhealthy weight. There were gaps in parental understanding of infant feeding and mobility, specifically regarding how to recognize fullness, active play ideas for different ages, as well as age-appropriate healthy eating. Parents also described being inundated with unsolicited advice about their child's diet or eating habits, causing uncertainty or anxiety.

We also found that parents of infants were open to obesity prevention intervention and guidance. When asked how they would prefer to receive anticipatory guidance about obesity risk during infancy, parents overwhelmingly identified pediatricians as the best, most trusted source of health information. This mirrors research in older populations (39). When prompted to think about how pediatricians should frame the conversation, it was clear that early life obesity is a sensitive topic. Our findings suggest that pediatricians initiate anticipatory guidance around obesity prevention at birth, building a relationship with parents over time, and focus conversations more on making practical changes to build healthy habits early than on obesity prevention *per se*. For example, while parents resisted the idea of “dieting” for their infants, they recognized the importance of nutrition and expressed interest in having specific, focused recommendations for healthy meals and snacks. Unlike most existing obesity prevention efforts that target mothers (40, 41), they recommended that information about how to make healthy changes engage non-maternal caregivers (e.g., fathers).

Parents expect providers to be explicit when there is a weight concern, but they also emphasized a preference for positivity and encouragement their approach. Parents discussed feeling defensive, personally attacked, hurt, embarrassed, offended, and upset by unsolicited advice about parenting and suggested that physicians be mindful of these possible reactions when providing anticipatory guidance about obesity risk. Our findings also suggest that tailoring interventions to the child's age, gender, and specific family dynamics is critical, as noted by others (42).

Despite the importance of obesity prevention, interventions delivered to infants tend to report low levels of parental engagement, as well as modest effects on children's weight outcomes (43–45). One key step to engaging parents in preventive efforts is improving their recognition of obesity risk (46, 47). However, evidence shows that mothers underestimate their infants' overweight (18, 38, 48), are uncertain whether infants are susceptible to being or becoming overweight (18), and may not be aware of risk factors during infancy that are important for obesity prevention (18). In other research, parents who have inaccurate perceptions of their child's obesity risk are more likely to ignore appropriate health messages (49). Some evidence further indicates that attempts by pediatricians to correct parents' misperceptions about child weight may damage rapport and ultimately fail if such messages fail to account for parent preferences (49). The findings from this study may help inform efforts to communicate obesity risk in the pediatric setting, a key step to developing effective interventions.

### Limitations

There were several limitations to this study. One limitation was the low numbers of fathers we were able to recruit. Further research should identify preferences and needs unique to fathers. We were not able to recruit the desired number of parents with incomes below $50,000 perhaps due to the online recruitment and engagement methods used. This may have limited the feedback and perspectives of those most affected by obesity and its comorbidities. We grouped participants by income level and child age to facilitate discussion, but participants' views were more homogenous than expected. Selection bias may have played a role, but we did not collect information on eligible parents who did not consent to participate. COVID-related stressors (e.g., food insecurity or feeding practices) (50) influenced parental responses, but we did not ask about this. Finally, our HCD approach, which provides a framework to understand the communication preferences and experiences of parents who are the target of obesity-prevention efforts, does not mimic traditional qualitative research approaches. The design methods we employed were primarily oriented toward generative solutions of defined problems within a small sample, rather than toward theory and hypotheses building as expected from more traditional research methods.

### Implications for Research and Practice

Implications for practice: Nonetheless, our HCD approach with the insight of parents successfully identified their perceptions, attitudes, and beliefs about risk of obesity in their young children. How we effectively communicate around obesity is an important topic as treatment paradigms shift more upstream toward prevention; our findings may be helpful in creating communication strategies to help parents and providers engage in these discussions. We learned that parents were receptive to prevention but were uncertain about early life obesity management, highlighting an opportunity for education. Encouragingly, parents were confident in their ability to make good choices for their children and, when uncertain, looked to their pediatrician for health advice. Our findings demonstrate the importance of a non-judgmental communication approach. Discussions should frame the conversation within the context of other developmental milestones, involve the entire family, and provide practical strategies for behavioral change.

Implications for research: Our findings support Matheson's et al. (51) call for design methodologies as research tools for chronic disease prevention. HCD, which provides a framework by which to understand the needs of stakeholders, may ultimately improve uptake, acceptability and usability of early life obesity interventions by ensuring that parents remain at the center of prevention efforts.

In conclusion, the findings of this study highlight important views of obesity risk and parental preferences regarding communication of risk for young infants. Our use of human-centered design releveled important opportunities to develop and refine messages and materials aimed at increasing parent/provider communication about early life obesity prevention.

## Data Availability Statement

The raw data supporting the conclusions of this article will be made available by the authors, without undue reservation.

## Ethics Statement

The studies involving human participants were reviewed and approved by Indiana University Institutional Review Board. The Ethics Committee waived the requirement of written informed consent for participation.

## Author Contributions

EC contributed to the conception and design of the study and wrote the first draft of the manuscript. CM and LP contributed to the design of the study, performed the analysis, and interpreted the results. ET, SW, and AC contributed to manuscript revision. All authors contributed to the article and approved the submitted version.

## Funding

This work was supported in part by NIH Grants K01DK114383 and K24DK10598. Research Jam: Indiana Clinical and Translational Sciences Institute's Patient Engagement Core (PEC) is supported by the National Center for Advancing Translational Sciences, Clinical and Translational Sciences Award [UL1TR002529]. No funder or sponsor had a role in the design and conduct of the study, collection, management, analysis, and interpretation of the data; preparation, review, or approval of the manuscript, or decision to submit the manuscript for publication.

## Conflict of Interest

The authors declare that the research was conducted in the absence of any commercial or financial relationships that could be construed as a potential conflict of interest.

## Publisher's Note

All claims expressed in this article are solely those of the authors and do not necessarily represent those of their affiliated organizations, or those of the publisher, the editors and the reviewers. Any product that may be evaluated in this article, or claim that may be made by its manufacturer, is not guaranteed or endorsed by the publisher.
